# Dibromido{2-[(4-nitro­phen­yl)imino­meth­yl]pyridine-κ^2^ *N*,*N*′}zinc(II)

**DOI:** 10.1107/S1600536811042231

**Published:** 2011-10-22

**Authors:** Sadegh Salehzadeh, Mehdi Khalaj, Saeed Dehghanpour, Isaac Tarmoradi

**Affiliations:** a Faculty of Chemistry, Bu-Ali Sina University, Hamedan, Iran; b Department of Chemistry, Alzahra University, PO Box 1993891176, Vanak, Tehran, Iran

## Abstract

In the title compound, [ZnBr_2_(C_12_H_9_N_3_O_2_)], the Zn^II^ ion is bonded to two Br ions and two N atoms of the diimine ligand in a distorted tetra­hedral geometry. With the exception of the Br atoms, all other atoms are disordered over two sets of sites corresponding to a 180° rotation of the mol­ecule along [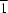
02]. The refined occupancies of the components are 0.809 (2) and 0.191 (2). In addition, the crystal studied was a non-merohedral twin with a refined component ratio of 0.343 (2):0.657 (2).

## Related literature

For related structures, see: Khalaj *et al.* (2009 ▶). For background information on diimine complexes, see: Khalaj *et al.* (2010 ▶); Salehzadeh *et al.* (2011 ▶).
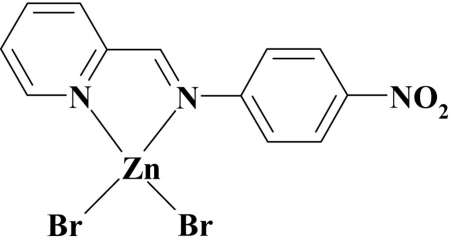


## Experimental

### 

#### Crystal data

[ZnBr_2_(C_12_H_9_N_3_O_2_)]
*M* *_r_* = 452.41
Triclinic, 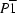

*a* = 7.2614 (5) Å
*b* = 7.9228 (8) Å
*c* = 13.6436 (15) Å
α = 87.724 (4)°
β = 74.719 (6)°
γ = 82.007 (6)°
*V* = 749.81 (12) Å^3^
*Z* = 2
Mo *K*α radiation
μ = 6.97 mm^−1^
*T* = 150 K
0.28 × 0.15 × 0.08 mm

#### Data collection

Nonius KappaCCD diffractometer
Absorption correction: multi-scan (*SORTAV*; Blessing, 1995 ▶) *T*_min_ = 0.417, *T*_max_ = 0.588
5868 measured reflections
3252 independent reflections
2630 reflections with *I* > 2σ(*I*)
*R*_int_ = 0.082

#### Refinement

*R*[*F* ^2^ > 2σ(*F* ^2^)] = 0.056
*wR*(*F* ^2^) = 0.140
*S* = 1.04
3252 reflections
237 parameters
48 restraints
H-atom parameters constrained
Δρ_max_ = 0.70 e Å^−3^
Δρ_min_ = −1.24 e Å^−3^

### 

Data collection: *COLLECT* (Nonius, 2002 ▶); cell refinement: *DENZO-SMN* (Otwinowski & Minor, 1997 ▶); data reduction: *DENZO-SMN*; program(s) used to solve structure: *SIR92* (Altomare *et al.*, 1994 ▶); program(s) used to refine structure: *SHELXTL* (Sheldrick, 2008 ▶); molecular graphics: *PLATON* (Spek, 2009 ▶); software used to prepare material for publication: *SHELXTL*.

## Supplementary Material

Crystal structure: contains datablock(s) I, global. DOI: 10.1107/S1600536811042231/gk2406sup1.cif

Structure factors: contains datablock(s) I. DOI: 10.1107/S1600536811042231/gk2406Isup2.hkl

Additional supplementary materials:  crystallographic information; 3D view; checkCIF report

## Figures and Tables

**Table:** 

Br1—Zn1	2.3428 (14)
Br2—Zn1	2.3357 (16)
Zn1—N1	2.034 (9)
Zn1—N2	2.074 (7)

**Table:** 

N1—Zn1—N2	81.2 (3)
N1—Zn1—Br2	116.1 (3)
N2—Zn1—Br2	118.0 (2)
N1—Zn1—Br1	112.3 (3)
N2—Zn1—Br1	111.5 (2)
Br2—Zn1—Br1	113.75 (5)

**Fig. 1.**
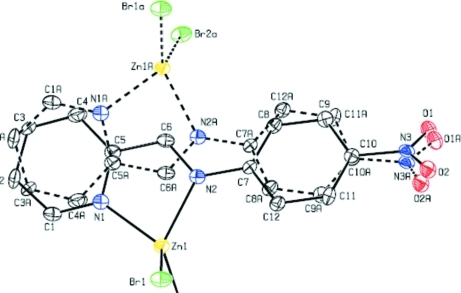
The molecular structure of the title compound showing both disordered components. The minor component is labeled with the suffix 'A'. The atoms Br1a and Br2a are related by the symmetry operation (x-1, y, x).

## supplementary crystallographic information

### Comment

In our ongoing studies on the synthesis, structural and spectroscopic characterization of transition metal complexes with diimine ligands Khalaj *et al.* (2010); Salehzadeh *et al.* (2011), we report herein the crystal structure of the title complex that was prepared by the reaction of ZnBr_2_ with the bidentate ligand (4-nitrophenyl)-pyridine-2-ylmethylene-amine (Scheme I).

The molecluar structure of the title complex is shown in Fig. 1. The Zn^II^ ion is in a distorted tetrahedral environment formed by the chelating ligand and two Br ions. A comparison of the dihedral angles between the planes of the pyridine, chelate and the benzene ring indicates that the ligand is distorted from planarity, with twist of 22.23 (24)° between the chelate (N1C5C6N2) and the benzene (C7C8C9C10C11C12) planes. The Zn—Br and Zn—N bond dimensions compare well with the values found in other tetrahedral diimine complexes of zinc bromide (Khalaj *et al.*, 2009).

### Experimental

The title complex was prepared by the reaction of ZnBr_2_ (22.5 mg, 0.1 mmol) and (4-nitrophenyl)pyridin-2-ylmethyleneamine (22.7 mg, 0.1 mmol) in 15 ml acetonitrile at room temperature. The solution was then concentrated under vacuum, and diffusion of diethyl ether vapor into the concentrated solution gave yellow crystals of the title compound in 60% yield.

### Refinement

The H(C) atom positions were calculated and refined in isotropic approximation within riding model with the *U*_iso_(H) parameters equal to 1.2 *U*_eq_(C) where U_eq_(C) is the equivalent thermal parameter of the carbon atoms to which corresponding H atoms are bonded. When the results of the initial refinements of the structure were examined for twinning the PLATON (Spek, 2009) software indicated that the crystal was a non-merohedral twin with twin matrix -1 0 0, 0 -1 0, -1 0 1. When refined using data generated by this twin matrix the ratio of the twin components refined to 0.342 (2): 0.658. Further to refinement of the twin components, residual electron density peaks were located in difference Fourier maps which indicated the structure was disordered. All atoms, except for the Br atoms were modeled as disordered corresponding to a rotation of approximately 180° (see Fig. 1). The Br atoms related by unit cell translations along the a axis are in sites which coordinate to both the major and minor components of disorder with an occupancy ratio of 0.809 (2):0.191 (2). The geometry of the twin components were constrained to be the same using the SAME instruction in SHELXL (Sheldrick, 2008) and the anisotropic displacement parameters of each individual major and minor atom site were constrained to be equal using the EADP instruction in SHELXL. The twin law corresponds to a 180° rotation about the [-1 0 2] direction and this direction is parallel to the rotation axis relating the two disordered sites of the molecule.

### Crystal data

**Table:**

[ZnBr_2_(C_12_H_9_N_3_O_2_)]	*Z* = 2
*M**_r_* = 452.41	*F*(000) = 436
Triclinic, *P*1	*D*_x_ = 2.004 Mg m^−^^3^
Hall symbol: -P 1	Mo *K*α radiation, λ = 0.71073 Å
*a* = 7.2614 (5) Å	Cell parameters from 6572 reflections
*b* = 7.9228 (8) Å	θ = 2.6–27.5°
*c* = 13.6436 (15) Å	µ = 6.97 mm^−^^1^
α = 87.724 (4)°	*T* = 150 K
β = 74.719 (6)°	Plate, colourless
γ = 82.007 (6)°	0.28 × 0.15 × 0.08 mm
*V* = 749.81 (12) Å^3^	

### Data collection

**Table:**

Nonius KappaCCD diffractometer	3252 independent reflections
Radiation source: fine-focus sealed tube	2630 reflections with *I* > 2σ(*I*)
graphite	*R*_int_ = 0.082
Detector resolution: 9 pixels mm^-1^	θ_max_ = 27.6°, θ_min_ = 2.6°
φ scans and ω scans with κ offsets	*h* = −9→9
Absorption correction: multi-scan (*SORTAV*; Blessing, 1995)	*k* = −10→10
*T*_min_ = 0.417, *T*_max_ = 0.588	*l* = −9→17
5868 measured reflections	

### Refinement

**Table:**

Refinement on *F*^2^	Primary atom site location: structure-invariant direct methods
Least-squares matrix: full	Secondary atom site location: difference Fourier map
*R*[*F*^2^ > 2σ(*F*^2^)] = 0.056	Hydrogen site location: inferred from neighbouring sites
*wR*(*F*^2^) = 0.140	H-atom parameters constrained
*S* = 1.04	*w* = 1/[σ^2^(*F*_o_^2^) + (0.0564*P*)^2^ + 2.802*P*] where *P* = (*F*_o_^2^ + 2*F*_c_^2^)/3
3252 reflections	(Δ/σ)_max_ < 0.001
237 parameters	Δρ_max_ = 0.70 e Å^−^^3^
48 restraints	Δρ_min_ = −1.24 e Å^−^^3^

### Special details

**Table:**

Geometry. All e.s.d.'s (except the e.s.d. in the dihedral angle between two l.s. planes) are estimated using the full covariance matrix. The cell e.s.d.'s are taken into account individually in the estimation of e.s.d.'s in distances, angles and torsion angles; correlations between e.s.d.'s in cell parameters are only used when they are defined by crystal symmetry. An approximate (isotropic) treatment of cell e.s.d.'s is used for estimating e.s.d.'s involving l.s. planes.
Refinement. Refinement of *F*^2^ against ALL reflections. The weighted *R*-factor *wR* and goodness of fit *S* are based on *F*^2^, conventional *R*-factors *R* are based on *F*, with *F* set to zero for negative *F*^2^. The threshold expression of *F*^2^ > σ(*F*^2^) is used only for calculating *R*-factors(gt) *etc*. and is not relevant to the choice of reflections for refinement. *R*-factors based on *F*^2^ are statistically about twice as large as those based on *F*, and *R*- factors based on ALL data will be even larger.

### Fractional atomic coordinates and isotropic or equivalent isotropic displacement parameters (Å^2^)

**Table:**

	*x*	*y*	*z*	*U*_iso_*/*U*_eq_	Occ. (<1)
Br1	0.33600 (13)	0.49862 (12)	0.72990 (8)	0.0358 (2)	
Br2	0.43682 (13)	−0.00007 (12)	0.71961 (8)	0.0366 (3)	
Zn1	0.20560 (17)	0.24153 (16)	0.74480 (11)	0.0259 (3)	0.809 (2)
O1	0.056 (2)	0.137 (2)	0.2086 (8)	0.036 (3)	0.809 (2)
O2	0.2084 (14)	0.3589 (19)	0.1962 (8)	0.034 (3)	0.809 (2)
N1	−0.0223 (12)	0.2369 (13)	0.8687 (7)	0.027 (2)	0.809 (2)
N2	−0.0123 (10)	0.2536 (11)	0.6714 (6)	0.0235 (16)	0.809 (2)
N3	0.1139 (15)	0.2531 (18)	0.2458 (5)	0.027 (2)	0.809 (2)
C1	−0.0292 (14)	0.2250 (15)	0.9678 (9)	0.032 (2)	0.809 (2)
H1A	0.0888	0.2123	0.9868	0.038*	0.809 (2)
C2	−0.2000 (15)	0.2302 (17)	1.0447 (9)	0.036 (3)	0.809 (2)
H2A	−0.1998	0.2239	1.1143	0.043*	0.809 (2)
C3	−0.3711 (15)	0.245 (2)	1.0150 (10)	0.038 (3)	0.809 (2)
H3A	−0.4906	0.2452	1.0647	0.046*	0.809 (2)
C4	−0.3672 (16)	0.259 (2)	0.9150 (10)	0.042 (3)	0.809 (2)
H4A	−0.4839	0.2762	0.8947	0.050*	0.809 (2)
C5	−0.1929 (19)	0.249 (2)	0.8435 (6)	0.029 (2)	0.809 (2)
C6	−0.1789 (12)	0.2637 (12)	0.7350 (8)	0.027 (2)	0.809 (2)
H6A	−0.2922	0.2807	0.7115	0.032*	0.809 (2)
C7	0.0079 (12)	0.2568 (13)	0.5649 (7)	0.022 (2)	0.809 (2)
C8	−0.1213 (16)	0.1964 (17)	0.5189 (8)	0.026 (2)	0.809 (2)
H8A	−0.2320	0.1533	0.5603	0.032*	0.809 (2)
C9	−0.091 (2)	0.198 (2)	0.4148 (11)	0.028 (3)	0.809 (2)
H9A	−0.1801	0.1597	0.3837	0.033*	0.809 (2)
C10	0.074 (3)	0.259 (2)	0.3573 (6)	0.023 (3)	0.809 (2)
C11	0.2013 (19)	0.3242 (17)	0.3996 (9)	0.024 (3)	0.809 (2)
H11A	0.3071	0.3731	0.3575	0.029*	0.809 (2)
C12	0.1737 (16)	0.3183 (16)	0.5044 (8)	0.027 (2)	0.809 (2)
H12A	0.2651	0.3551	0.5345	0.032*	0.809 (2)
Zn1A	−0.4500 (7)	0.2540 (7)	0.7425 (4)	0.0259 (3)	0.191 (2)
O1A	0.101 (12)	0.141 (12)	0.196 (3)	0.036 (3)	0.191 (2)
O2A	0.252 (8)	0.354 (10)	0.211 (3)	0.034 (3)	0.191 (2)
N1A	−0.349 (2)	0.259 (6)	0.8676 (10)	0.027 (2)	0.191 (2)
N2A	−0.1587 (13)	0.243 (4)	0.6709 (11)	0.0235 (16)	0.191 (2)
N3A	0.156 (7)	0.240 (8)	0.2468 (11)	0.027 (2)	0.191 (2)
C1A	−0.441 (3)	0.265 (6)	0.9666 (11)	0.032 (2)	0.191 (2)
H1AA	−0.5778	0.2718	0.9850	0.038*	0.191 (2)
C2A	−0.348 (4)	0.262 (8)	1.0440 (14)	0.036 (3)	0.191 (2)
H2AA	−0.4183	0.2797	1.1130	0.043*	0.191 (2)
C3A	−0.147 (4)	0.233 (9)	1.0156 (16)	0.038 (3)	0.191 (2)
H3AA	−0.0783	0.2113	1.0660	0.046*	0.191 (2)
C4A	−0.051 (3)	0.234 (10)	0.916 (2)	0.042 (3)	0.191 (2)
H4AA	0.0856	0.2229	0.8961	0.050*	0.191 (2)
C5A	−0.154 (3)	0.252 (7)	0.8437 (12)	0.029 (2)	0.191 (2)
C6A	−0.0591 (17)	0.252 (5)	0.7351 (15)	0.027 (2)	0.191 (2)
H6AA	0.0752	0.2576	0.7121	0.032*	0.191 (2)
C7A	−0.072 (2)	0.246 (4)	0.5645 (11)	0.022 (2)	0.191 (2)
C8A	0.108 (4)	0.298 (7)	0.5209 (16)	0.026 (2)	0.191 (2)
H8AA	0.1774	0.3375	0.5635	0.032*	0.191 (2)
C9A	0.187 (6)	0.292 (10)	0.4172 (18)	0.028 (3)	0.191 (2)
H9AA	0.3085	0.3294	0.3875	0.033*	0.191 (2)
C10A	0.083 (8)	0.231 (11)	0.3581 (11)	0.023 (3)	0.191 (2)
C11A	−0.090 (8)	0.169 (11)	0.3986 (17)	0.024 (3)	0.191 (2)
H11B	−0.1520	0.1210	0.3556	0.029*	0.191 (2)
C12A	−0.171 (5)	0.178 (8)	0.5029 (14)	0.027 (2)	0.191 (2)
H12B	−0.2923	0.1394	0.5319	0.032*	0.191 (2)

### Atomic displacement parameters (Å^2^)

**Table:**

	*U*^11^	*U*^22^	*U*^33^	*U*^12^	*U*^13^	*U*^23^
Br1	0.0307 (5)	0.0262 (4)	0.0540 (6)	−0.0087 (3)	−0.0146 (5)	−0.0002 (5)
Br2	0.0281 (4)	0.0272 (4)	0.0537 (7)	−0.0054 (3)	−0.0079 (5)	−0.0030 (5)
Zn1	0.0226 (5)	0.0275 (5)	0.0292 (6)	−0.0083 (4)	−0.0068 (5)	−0.0006 (6)
O1	0.041 (9)	0.039 (4)	0.031 (5)	−0.007 (6)	−0.013 (4)	−0.010 (4)
O2	0.037 (7)	0.035 (4)	0.029 (5)	−0.010 (5)	−0.009 (4)	0.009 (4)
N1	0.028 (4)	0.024 (4)	0.031 (5)	−0.005 (3)	−0.008 (4)	0.001 (5)
N2	0.026 (4)	0.016 (4)	0.031 (4)	−0.004 (3)	−0.009 (4)	−0.004 (4)
N3	0.023 (6)	0.029 (4)	0.031 (4)	0.006 (5)	−0.013 (4)	−0.003 (5)
C1	0.026 (5)	0.032 (5)	0.040 (7)	−0.001 (4)	−0.013 (5)	0.003 (6)
C2	0.042 (6)	0.043 (7)	0.017 (5)	−0.007 (6)	0.003 (5)	−0.006 (6)
C3	0.018 (5)	0.065 (9)	0.028 (7)	−0.005 (5)	0.002 (5)	−0.008 (7)
C4	0.023 (5)	0.060 (8)	0.042 (8)	−0.010 (5)	−0.006 (6)	0.012 (8)
C5	0.026 (6)	0.029 (4)	0.034 (5)	−0.009 (5)	−0.008 (5)	−0.009 (5)
C6	0.023 (4)	0.023 (5)	0.037 (6)	−0.006 (4)	−0.011 (5)	0.001 (5)
C7	0.020 (6)	0.020 (4)	0.024 (4)	−0.002 (5)	−0.002 (4)	0.001 (4)
C8	0.025 (6)	0.022 (5)	0.030 (6)	−0.009 (4)	−0.001 (5)	0.000 (5)
C9	0.031 (6)	0.019 (8)	0.033 (7)	−0.004 (5)	−0.008 (5)	−0.007 (5)
C10	0.025 (4)	0.015 (8)	0.027 (4)	0.003 (4)	−0.006 (4)	0.001 (4)
C11	0.033 (6)	0.014 (6)	0.021 (6)	−0.005 (4)	0.001 (5)	0.002 (4)
C12	0.025 (6)	0.027 (5)	0.029 (6)	−0.011 (5)	−0.003 (4)	−0.001 (5)
Zn1A	0.0226 (5)	0.0275 (5)	0.0292 (6)	−0.0083 (4)	−0.0068 (5)	−0.0006 (6)
O1A	0.041 (9)	0.039 (4)	0.031 (5)	−0.007 (6)	−0.013 (4)	−0.010 (4)
O2A	0.037 (7)	0.035 (4)	0.029 (5)	−0.010 (5)	−0.009 (4)	0.009 (4)
N1A	0.028 (4)	0.024 (4)	0.031 (5)	−0.005 (3)	−0.008 (4)	0.001 (5)
N2A	0.026 (4)	0.016 (4)	0.031 (4)	−0.004 (3)	−0.009 (4)	−0.004 (4)
N3A	0.023 (6)	0.029 (4)	0.031 (4)	0.006 (5)	−0.013 (4)	−0.003 (5)
C1A	0.026 (5)	0.032 (5)	0.040 (7)	−0.001 (4)	−0.013 (5)	0.003 (6)
C2A	0.042 (6)	0.043 (7)	0.017 (5)	−0.007 (6)	0.003 (5)	−0.006 (6)
C3A	0.018 (5)	0.065 (9)	0.028 (7)	−0.005 (5)	0.002 (5)	−0.008 (7)
C4A	0.023 (5)	0.060 (8)	0.042 (8)	−0.010 (5)	−0.006 (6)	0.012 (8)
C5A	0.026 (6)	0.029 (4)	0.034 (5)	−0.009 (5)	−0.008 (5)	−0.009 (5)
C6A	0.023 (4)	0.023 (5)	0.037 (6)	−0.006 (4)	−0.011 (5)	0.001 (5)
C7A	0.020 (6)	0.020 (4)	0.024 (4)	−0.002 (5)	−0.002 (4)	0.001 (4)
C8A	0.025 (6)	0.022 (5)	0.030 (6)	−0.009 (4)	−0.001 (5)	0.000 (5)
C9A	0.031 (6)	0.019 (8)	0.033 (7)	−0.004 (5)	−0.008 (5)	−0.007 (5)
C10A	0.025 (4)	0.015 (8)	0.027 (4)	0.003 (4)	−0.006 (4)	0.001 (4)
C11A	0.033 (6)	0.014 (6)	0.021 (6)	−0.005 (4)	0.001 (5)	0.002 (4)
C12A	0.025 (6)	0.027 (5)	0.029 (6)	−0.011 (5)	−0.003 (4)	−0.001 (5)

### Geometric parameters (Å, °)

**Table:**

Br1—Zn1A^i^	2.340 (5)	C12—H12A	0.9500
Br1—Zn1	2.3428 (14)	Zn1A—N1A	2.033 (9)
Br2—Zn1	2.3357 (16)	Zn1A—N2A	2.074 (8)
Br2—Zn1A^i^	2.339 (5)	Zn1A—Br2^ii^	2.339 (5)
Zn1—N1	2.034 (9)	Zn1A—Br1^ii^	2.340 (5)
Zn1—N2	2.074 (7)	O1A—N3A	1.239 (10)
O1—N3	1.239 (10)	O2A—N3A	1.226 (10)
O2—N3	1.226 (10)	N1A—C1A	1.340 (14)
N1—C1	1.340 (14)	N1A—C5A	1.361 (16)
N1—C5	1.361 (16)	N2A—C6A	1.284 (11)
N2—C6	1.284 (11)	N2A—C7A	1.422 (12)
N2—C7	1.421 (12)	N3A—C10A	1.472 (10)
N3—C10	1.472 (10)	C1A—C2A	1.394 (14)
C1—C2	1.394 (14)	C1A—H1AA	0.9500
C1—H1A	0.9500	C2A—C3A	1.394 (15)
C2—C3	1.394 (15)	C2A—H2AA	0.9500
C2—H2A	0.9500	C3A—C4A	1.357 (18)
C3—C4	1.357 (18)	C3A—H3AA	0.9500
C3—H3A	0.9500	C4A—C5A	1.373 (16)
C4—C5	1.373 (16)	C4A—H4AA	0.9500
C4—H4A	0.9500	C5A—C6A	1.459 (14)
C5—C6	1.459 (14)	C6A—H6AA	0.9500
C6—H6A	0.9500	C7A—C8A	1.400 (14)
C7—C8	1.400 (14)	C7A—C12A	1.406 (12)
C7—C12	1.405 (12)	C8A—C9A	1.379 (18)
C8—C9	1.379 (18)	C8A—H8AA	0.9500
C8—H8A	0.9500	C9A—C10A	1.381 (18)
C9—C10	1.381 (18)	C9A—H9AA	0.9500
C9—H9A	0.9500	C10A—C11A	1.380 (19)
C10—C11	1.380 (19)	C11A—C12A	1.390 (16)
C11—C12	1.390 (16)	C11A—H11B	0.9500
C11—H11A	0.9500	C12A—H12B	0.9500
			
Zn1A^i^—Br1—Zn1	64.77 (11)	C7—C12—H12A	120.6
Zn1—Br2—Zn1A^i^	64.90 (12)	N1A—Zn1A—N2A	81.2 (3)
N1—Zn1—N2	81.2 (3)	N1A—Zn1A—Br2^ii^	114.8 (13)
N1—Zn1—Br2	116.1 (3)	N2A—Zn1A—Br2^ii^	110.8 (8)
N2—Zn1—Br2	118.0 (2)	N1A—Zn1A—Br1^ii^	111.9 (12)
N1—Zn1—Br1	112.3 (3)	N2A—Zn1A—Br1^ii^	120.6 (8)
N2—Zn1—Br1	111.5 (2)	Br2^ii^—Zn1A—Br1^ii^	113.73 (19)
Br2—Zn1—Br1	113.75 (5)	C1A—N1A—C5A	116.9 (9)
C1—N1—C5	117.0 (8)	C1A—N1A—Zn1A	130.7 (7)
C1—N1—Zn1	130.6 (7)	C5A—N1A—Zn1A	112.4 (7)
C5—N1—Zn1	112.4 (7)	C6A—N2A—C7A	121.1 (8)
C6—N2—C7	121.2 (7)	C6A—N2A—Zn1A	111.5 (6)
C6—N2—Zn1	111.5 (6)	C7A—N2A—Zn1A	127.0 (6)
C7—N2—Zn1	127.2 (5)	O2A—N3A—O1A	124.4 (8)
O2—N3—O1	124.5 (7)	O2A—N3A—C10A	117.9 (9)
O2—N3—C10	118.0 (9)	O1A—N3A—C10A	117.4 (9)
O1—N3—C10	117.5 (9)	N1A—C1A—C2A	123.4 (10)
N1—C1—C2	123.6 (10)	N1A—C1A—H1AA	118.3
N1—C1—H1A	118.2	C2A—C1A—H1AA	118.3
C2—C1—H1A	118.2	C1A—C2A—C3A	117.0 (12)
C1—C2—C3	117.2 (12)	C1A—C2A—H2AA	121.5
C1—C2—H2A	121.4	C3A—C2A—H2AA	121.5
C3—C2—H2A	121.4	C4A—C3A—C2A	119.8 (12)
C4—C3—C2	120.0 (11)	C4A—C3A—H3AA	120.1
C4—C3—H3A	120.0	C2A—C3A—H3AA	120.1
C2—C3—H3A	120.0	C3A—C4A—C5A	119.3 (11)
C3—C4—C5	119.4 (11)	C3A—C4A—H4AA	120.4
C3—C4—H4A	120.3	C5A—C4A—H4AA	120.4
C5—C4—H4A	120.3	N1A—C5A—C4A	122.7 (10)
N1—C5—C4	122.6 (10)	N1A—C5A—C6A	115.1 (9)
N1—C5—C6	115.1 (9)	C4A—C5A—C6A	122.1 (12)
C4—C5—C6	122.1 (11)	N2A—C6A—C5A	119.5 (9)
N2—C6—C5	119.5 (9)	N2A—C6A—H6AA	120.2
N2—C6—H6A	120.2	C5A—C6A—H6AA	120.2
C5—C6—H6A	120.2	C8A—C7A—C12A	119.9 (9)
C8—C7—C12	119.9 (8)	C8A—C7A—N2A	123.9 (8)
C8—C7—N2	123.9 (8)	C12A—C7A—N2A	116.0 (8)
C12—C7—N2	116.1 (8)	C9A—C8A—C7A	121.2 (10)
C9—C8—C7	121.2 (10)	C9A—C8A—H8AA	119.4
C9—C8—H8A	119.4	C7A—C8A—H8AA	119.4
C7—C8—H8A	119.4	C8A—C9A—C10A	117.6 (12)
C8—C9—C10	117.6 (11)	C8A—C9A—H9AA	121.2
C8—C9—H9A	121.2	C10A—C9A—H9AA	121.2
C10—C9—H9A	121.2	C11A—C10A—C9A	122.9 (9)
C11—C10—C9	122.9 (8)	C11A—C10A—N3A	118.6 (12)
C11—C10—N3	118.7 (12)	C9A—C10A—N3A	118.4 (13)
C9—C10—N3	118.4 (13)	C10A—C11A—C12A	119.5 (10)
C10—C11—C12	119.4 (9)	C10A—C11A—H11B	120.3
C10—C11—H11A	120.3	C12A—C11A—H11B	120.3
C12—C11—H11A	120.3	C11A—C12A—C7A	118.7 (10)
C11—C12—C7	118.7 (10)	C11A—C12A—H12B	120.6
C11—C12—H12A	120.6	C7A—C12A—H12B	120.6

Symmetry codes: (i) *x*+1, *y*, *z*; (ii) *x*−1, *y*, *z*.
